# Immune Responses and Efficacy of *Brucella* Abortus Strain RB51 in Bison After Delivery in a Dry Dart Formulation or by Parenteral Inoculation

**DOI:** 10.3389/fvets.2021.706160

**Published:** 2021-07-30

**Authors:** Steven C. Olsen, Paola M. Boggiatto, Pauline Nol, Matthew P. McCollum, Jack C. Rhyan

**Affiliations:** ^1^Department of Agriculture, Infectious Bacterial Diseases of Livestock Research Unit, National Animal Disease Center, Agricultural Research Service, Ames, IA, United States; ^2^Wildlife Health Program, Colorado Division of Parks and Wildlife, Denver, CO, United States; ^3^Animal Reproduction and Biotechnology Lab, Colorado State University, Fort Collins, CO, United States; ^4^Independent Consultant, Fort Collins, CO, United States

**Keywords:** brucella, bison, vaccination, immunology, efficacy, remote vaccination

## Abstract

Bison (*Bison bison*) heifer calves (n = 32) were randomly assigned to control or vaccination with 10^10^ colony-forming units of *Brucella abortus* strain RB51 (RB51) vaccine by single or boostered parenteral delivery, or by surgical implantation of a dry dart formulation (*n* = 8/trt). Serum and/or peripheral blood mononuclear cells (PBMC) were obtained at 0, 4, 8, 13, 16, 21, and 24 wks after initial vaccination and at 0, 4, 8, 12, 15, 22, and 27 wks after booster vaccination to characterize humoral and cellular immune responses to RB51. Bison in both RB51 vaccination treatments demonstrated greater (*P* < 0.0001) serum humoral responses when compared to non-vaccinates, with parenteral vaccinates demonstrating greater (*P* < 0.01) responses when compared to mean responses of bison inoculated with the dry dart. Only the booster vaccinated treatment demonstrated greater (*P* < 0.0001) humoral responses than control bison in samples collected after re-inoculation. At 4, 8, 12, 16, and 24 wks after initial vaccination, PBMC from parenteral RB51 vaccinates demonstrated greater proliferative responses to RB51 when compared to responses of control animals. In comparison, bison inoculated with the RB51 dry dart did not demonstrate greater (*P* > 0.05) proliferative responses when compared to responses of non-vaccinates. Bison were pasture bred and pregnant animals experimentally challenged in mid-gestation with 10^7^ CFU of *B. abortus* strain 2,308. Bison in parenteral vaccination treatments had reduced (*P* < 0.05) abortions and infection in uterine and fetal samples as compared to non-vaccinated bison, with booster vaccinates tending to have the lowest colonization (CFU/gm) in tissues. In comparison, the dry dart formulation did reduce abortion (*P* < 0.05) but not infection (*P* > 0.05) in most tissues when compared to non-vaccinated bison. The results of this study reaffirm the efficacy of boostered parenteral vaccination of bison with RB51 in preventing brucellosis. Our data also suggests that the novel dry dart RB51 formulation does not induce sufficient efficacy in bison after a single inoculation.

## Introduction

From its beginning 90 years ago as a control program to reduce brucellosis prevalence during drought conditions, the United States national program has essentially eradicated brucellosis from domestic cattle herds. However, the maintenance of *Brucella abortus* infections in wildlife reservoirs, particularly in the Greater Yellowstone Area (GYA; Yellowstone National Park and surrounding areas), pose a risk for reintroduction of brucellosis to domestic livestock. Free-ranging elk (*Cervus canadensis*) have been implicated by epidemiologic investigations to most brucellosis infections in cattle over the past 20 years (1), including 5 herds in 2019 and 2020. Others have demonstrated that bison (*Bison bison*) can effectively transmit brucellosis to cattle when co-housed together (2). Current management actions in the GYA based on temporal and spatial separation are used to prevent potential brucellosis transmission between bison and cattle.

The bison herd in Yellowstone National Park is of historic, cultural and genetic importance. The National Park Service recognizes the herd as the largest bison population on public land and the only place where bison have lived continuously since prehistoric times. The Yellowstone herd is also important as one of the few herds that do not demonstrate evidence of introgression of cattle genes (3). Many bison producers are interested in improving their herds by introducing genetics of Yellowstone bison into their breeding programs, but brucellosis regulations negatively impact movement of bison from the herd. Epidemiologic data and historical records suggest that brucellosis was introduced into the Yellowstone bison by movement of cattle into the region (4). As brucellosis in bison can be described as an invasive species, elimination of the disease would be beneficial in restoring native environment conditions for bison. The National Park Service currently has programs for reducing and eliminating invasive species as part of efforts to maintain the natural biodiversity of national parks. Eliminating brucellosis in wildlife in the GYA area would also provide social and economic benefits by eliminating the need for expensive programs to prevent brucellosis transmission to livestock and perhaps allowing greater flexibility in management of wildlife resources.

One of the most effective tools for disease control is use of vaccines that induce protective immunity. We have previously demonstrated that parenteral vaccination with *B. abortus* strain RB51 (RB51), particularly after booster vaccination, is efficacious in preventing abortion or infection in bison after experimental challenge (5, 6). As a vaccination program to reduce disease prevalence in bison would require a long-term commitment and significant economic costs, maximizing the efficacy of the planned vaccine is essential. Although a remotely delivered vaccine would be required for delivery to free-ranging bison, vaccine formulation and delivery method may influence immune responses and efficacy (7). In the current study, we evaluated a novel dry dart formulation which incorporates *B. abortus* strain RB51 vaccine (RB51) in a delivery form amiable to remote delivery options. The objective was to determine if this formulation induces protective immunity in bison. To maximize immune responses in this pilot study, the dry dart pellet was delivered surgically instead of as a projectile.

## Methods

All experiments were approved by the National Animal Disease Center Animal Care and Use committee prior to initiation and conducted in accordance with national animal welfare regulations.

### Vaccine and Bacterial Cultures

The RB51 vaccine was obtained from a commercial source (Colorado Serum Co, Denver, CO). For serologic and proliferation assays, RB51 and *Brucella abortus* strain 2308 (S2308) was obtained from the culture repository at the National Animal Disease Center, Ames, IA. After growth on tryptose agar plates for 72 hr at 37°C and 5% CO_2_, RB51 and S2308 suspensions were removed from plates by washing with 0.15 sodium chloride (saline), concentrated by centrifugation, and inactivated by γ-irradiation (1.4 × 10^6^ rads). Following irradiation, suspensions were washed in saline and stored in 1 ml aliquots at −70°C until use.

### Dry Dart Preparation

Pellets were prepared by mixing 10 mg magnesium stearate (for lubrication) with lyophilized RB51 from two 25 dose bottles obtained from a commercial source (Colorado Serum Co., Denver, CO, USA). The powder mixture was compressed using a manual pellet making device into projectiles of 3 mm diameter x approximately 10 mm length with each pellet weighing between 118 and 144 mg each.

### Vaccination

Thirty-two bison heifers approximately 6 to 9 months of age were obtained from a brucellosis-free herd. One-fourth of the heifers (*n* = 8/treatment) were randomly assigned to the following treatments: (1) control; (2) single vaccination with 10^10^ colony-forming units (CFU) of the commercial RB51 vaccine; (3) parenteral vaccination with the commercial RB51 vaccine as calves combined with booster vaccination 10 months later with a similar dosage of RB51; and (4) surgical implantation of a dry dart formulation containing RB51. The parental RB51 vaccine was prepared in accordance with manufacturer's instructions and subcutaneously administered in the cervical region. The RB51 dry dart formulation was administered by making a small incision into the hide in the cervical region between the shoulder and head, inserting the dart approximately 4 cm into subcutaneous tissue, and delivery of the vaccine materials by plunger. The incision was closed with non-absorbable suture.

The concentration of RB51 in the inoculums were determined by dilution of the vaccines in saline and standard plate counts.

### Post-vaccination Serologic Responses

Blood samples were collected by jugular venipuncture prior to vaccination, and at 4, 8, 13, 16, 21, and 24 wks post-inoculation. Blood was also collected from all animals prior to administration of the RB51 booster inoculation, and at 4, 8, 12, 15, 22, and 27 wks after vaccination. Blood was allowed to clot for 12 hr at 4°C and centrifuged at 800 × g for 30 minutes. Serum was divided into 1 ml aliquots, frozen, and stored at −70°C. Antibody titers to *Brucella* were determined by a previously described ELISA in which γ-irradiated RB51 is used as antigen (8).

### Post-vaccination Lymphocyte Proliferation

At 4, 8, 13, 16, 21, and 24 wks after initial vaccination, and 12, 15, 22, and 27 weeks after booster vaccination, blood was obtained from the jugular vein of all bison and placed into an acid-citrate dextrose solution. Peripheral blood mononuclear cells (PBMC) were enriched by density centrifugation (Sigma Diagnostics, Inc., St. Louis, MO, USA) and adjusted to 110^7^ viable cells per ml in RPMI as determined by trypan blue dye exclusion.

Fifty μl of each cell suspension, containing 5 × 10^5^ cells, were added to each of two separate flat-bottom wells of 96-well microtiter plates that contained 100 μl of RPMI 1,640 medium only, or 1,640 medium containing γ-irradiated RB51 (10^5^ to 10^9^ bacteria per well). Cell cultures were incubated for 7 days at 37°C in 5% CO_2_. After 7 days incubation, cell cultures were pulsed with 1.0 μCi of [^3^H]-thymidine per well for 18 hr. Cells were harvested onto glass filter mats and counted for radioactivity in a liquid scintillation counter (Perkin Elmer, Hopkinton, MA, USA).

### Experimental *B. abortus* Challenge

Bison heifers were pasture bred at approximately 26 months of age and pregnancy status and stage of gestation determined by rectal palpation. Pregnant bison were transferred to a Biosafety level 3 containment facility at 2 to 3 weeks prior to challenge where they were housed for the duration of the study. Based on palpation data, pregnant bison were intraconjuctivally challenged between 170 and 180 days of gestation with approximately 1 × 10^7^ CFU of *B. abortus* strain 2,308 (50 μl of inoculum per eye). Concentration of bacteria within the challenge inoculum was determined by standard plate counts.

### Post-challenge Serology

Blood samples were collected by jugular venipuncture prior to experimental challenge, at 4 weeks post-challenge, and at necropsy after parturition or abortion. Blood was prepared as described previously and antibody responses to *Brucella* were determined by ELISA using γ-irradiated 2,308 as antigen (8).

### Necropsy Procedures

Bison were euthanized within 72 hr after parturition by intravenous injection of sodium pentobarbitol (Sleepaway, Ft. Dodge Labs, Ft. Dodge, IA, USA). Fetal viability was assessed, and crown-to-rump length of the fetus was recorded. Maternal samples obtained at necropsy for bacteriologic evaluation included: lymphatic tissues (bronchial, hepatic, internal iliac, mandibular, messenteric, parotid, prescapular, retropharyngeal, and suprammamary), samples of milk and mammary tissue from all four quarters, maternal placentome and/or caruncle, spleen, liver, lung and vaginal and conjunctival swabs. Samples obtained from calves included: rectal swab, lung, liver, spleen, bronchial lymph node, and abomasal contents. Fetal and maternal blood was also obtained at necropsy for bacteriologic and serologic evaluation and mixed 1:1 with tryptose broth (Difco Laboratories, Detroit, MI, USA) containing 1% sodium citrate.

### Bacterial Culture

For bacterial culture, tissues were triturated in 0.85% NaCl (saline) using a tissue grinder and placed on tryptose agar containing 5% bovine serum or Kuzdas and Morse plates (9) as previously described (6, 10, 11). Swabs were directly plated on tryptose agar plates containing 5% bovine serum. One ml from blood cultures of each heifer was directly plated on tryptose agar containing 5% bovine serum. Remaining blood cultures were held at −5° C for 24 hr and then placed at 37°C and 5% CO_2_ with 1 ml volumes plated onto tryptose agar containing 5% bovine serum after 7, 14, 21, and 28 days incubation. Following incubation at 37° C and 5% CO_2_ for 72 hr, *B. abortus* was identified on the basis of colony morphology, and growth characteristics (9). Isolation of *B. abortus* was confirmed using a polymerase chain reaction procedure designed to identify individual species of *Brucella* (12).

### Definitions

Abortion was defined as the premature birth of a *Brucella*-infected, non-viable fetus at any time after *B. abortus* challenge. Mammary infection was defined as the recovery of the *B. abortus* from milk, mammary gland, or supramammary lymph node, whereas uterine infection was defined as recovery from placentome, vaginal swab, or internal iliac lymph node. Maternal or fetal infection was defined as the recovery of *B. abortus* from any maternal or fetal sample, respectively.

### Statistical Analysis

Bacteriologic and proliferation data were converted to log_10_ with any values of 0 changed to 1 before transformation. Serologic, lymphoproliferative responses, and colonization data were compared by two-way general linear model procedure using treatment and time of sampling in the model, with means separated by a least square means procedure (SAS Institute Inc., Cary, NC, USA). Fisher's exact test was used to compare the rate of infection and abortion in vaccinates and controls after experimental challenge. Data are presented as mean ± SEM. Mean responses were considered significant when the statistical model indicated that *P* < 0.05.

## Results

### Vaccination and Challenge Inoculum Concentrations

Standard plate counts indicated that initial vaccines contained 1.8 × 10^10^ CFU for the commercial RB51 vaccine and dry dart pellets contained 9.9 × 10^9^ CFU of RB51. In a similar manner, the booster vaccine delivered 10 months after initial vaccination contained 1.1 × 10^10^ CFU.

Mean concentration of *B. abortus* in the experimental challenge inoculum was 0.98 × 10^7^ ± 0.15.

### Serologic Responses to Vaccination

**Figure 1:**
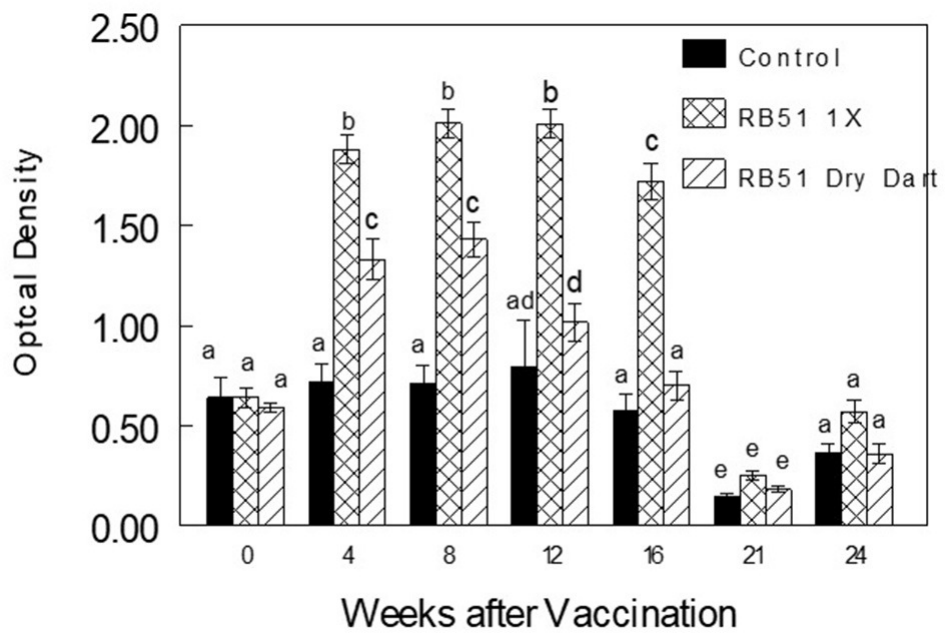
Serologic responses of bison to γ-irradiated *B. abortus* strain RB51 (RB51) in an ELISA assay in non-vaccinated (Control) or after vaccination with 10^10^ CFU of RB51 parenterally (RB51 1X) or in a novel dry dart formulation (RB51 Dry Dart). Responses are presented as mean titer ± SEM. Means with different superscripts are significantly different (*P* < 0.05).

After initial inoculation with the commercial RB51 vaccine or the RB51 dry dart, vaccinates in both treatments demonstrated greater (*P* < 0.0001) serum humoral responses in an ELISA assay to RB51 antigens when compared to mean responses of non-vaccinates (Figure 1). Bison vaccinated with the RB51 dry dart had greater (*P* < 0.0001) mean optical densities at 4 and 8 weeks when compared to responses of control bison. In comparison, parentally vaccinated bison had greater (*P* < 0.001) mean humoral responses at 4, 8, 12, and 16 weeks when compared to responses of non-vaccinated bison. Mean responses of parenteral vaccinates were greater (*P* < 0.01) at 4, 8, 12, and 16 weeks when compared to mean optical density of sera from bison inoculated with the dry dart.

**Figure 2:**
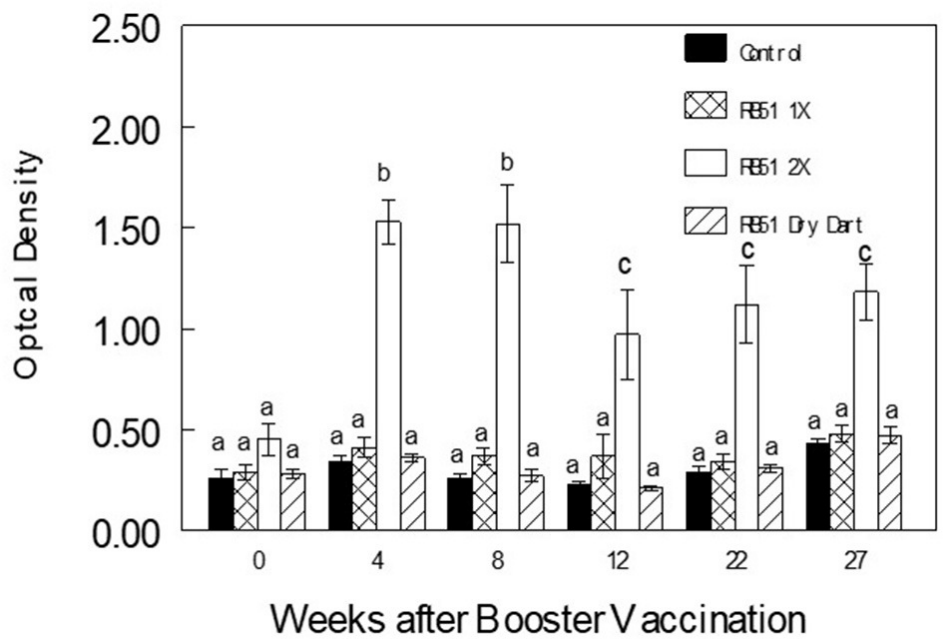
Serologic responses of bison to γ-irradiated *B. abortus* strain RB51 (RB51) in an ELISA assay after administration of 10^10^ CFU of RB51 to bison in the boostered vaccine treatment (RB51 2X) approximately 10 months after initial vaccination. Other treatments included non-vaccinated bison (Control), bison parenterally inoculated with a single dose of 10^10^ CFU of RB51 ten months previously (RB51 1X), and bison inoculated with a novel RB51 dry dart formulation 10 months previously (RB51 Dry Dart). Responses are presented as mean titer ± SEM. Means with different superscripts are significantly different (*P* < 0.05).

Prior to booster vaccination at ten months after initial vaccination, mean humoral responses to RB51 did not differ (*P* > 0.05) between any treatment (Figure 2). After parenteral administration of the RB51 booster vaccination, bison in this treatment demonstrated greater (*P* < 0.0001) mean serum responses on the ELISA assay at all sampling times after booster vaccination when compared to all other treatments (Figure 2).

### Lymphocyte Proliferation Assays

**Figure 3:**
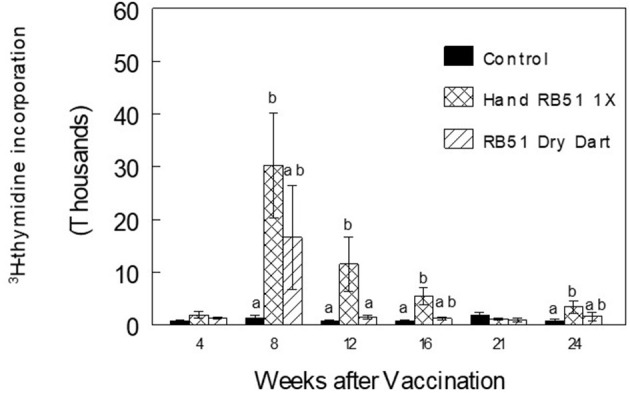
Proliferative responses to 10^7^ CFU of γ-irradiated RB51 by peripheral blood mononuclear cells from non-vaccinated bison (Control) or bison vaccinated with 10^10^ CFU of RB51 parenterally or in a novel dry dart formulation. Cells were incubated at 37° C and 5% CO_2_ for 7 days and pulsed for 18 hrs with [^3^H]-thymidine. Results are expressed as mean stimulation indexes ± SEM. Means within a sampling time with different superscripts are significantly different (*P* < 0.05).

After initial vaccination, PBMC from bison parenterally vaccinated with RB51 had greater (*P* < 0.05) mean antigen-specific proliferative responses at 8, 12, and 16 wks when compared to responses of non-vaccinates (Representative data in Figure 3). In comparison, PBMC from bison inoculated with the RB51 dry dart did not differ (*P* > 0.05) in proliferative responses to RB51 antigens at any sampling time when compared to responses of non-vaccinates. After initial vaccination, mean proliferative responses of bison parenterally vaccinated with RB51 were greater (*P* < 0.05) only at 12 wks when compared to mean responses of animals in the dry dart treatment.

After booster vaccination, antigen-specific proliferative responses of PBMC from booster vaccinates did not differ (*P* > 0.05) at any sampling time from mean responses of all other treatments (Data not shown).

### Pregnant Bison for Experimental Challenge

Rectal palpation indicated 7 bison were pregnant in the control and single RB51 vaccination groups, 5 bison in the boostered RB51 vaccine group, and 6 bison in the RB51 dry dart treatment.

### Serologic Responses After Experimental Challenge

**Figure 4:**
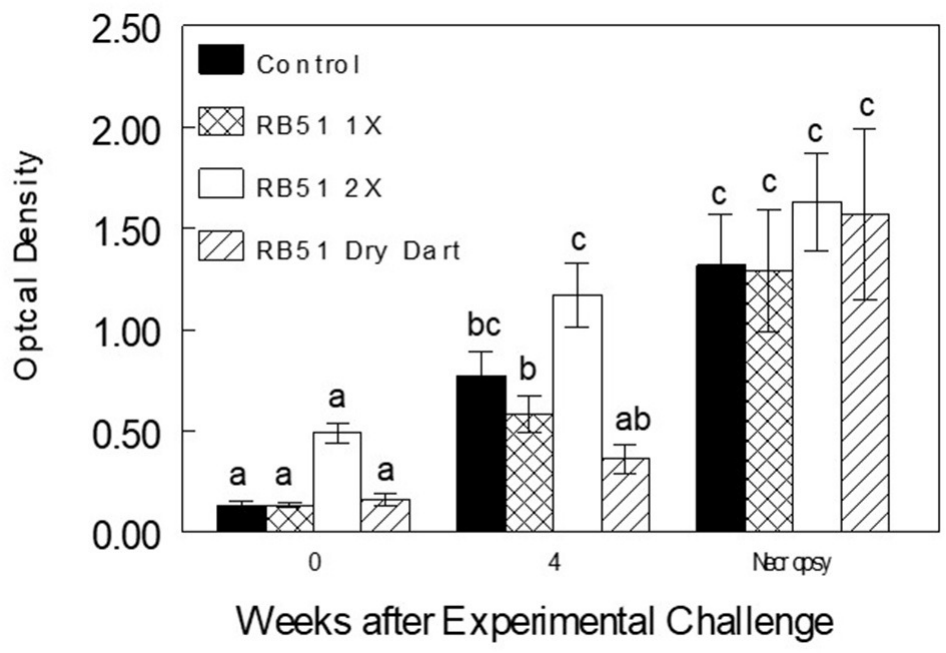
Serologic responses of bison to γ-irradiated *B. abortus* strain 2308 (2308) in an ELISA assay after experimental challenge in mid-gestation with 10^7^ CFU of 2308. Bison treatments included non-vaccinated (Control), or vaccination with 10^10^ CFU of *B. abortus* strain RB51 (RB51) in a single parenteral inoculation (RB51 1X), a boostered parenteral inoculation (RB51 2X), or in a novel dry dart formulation (RB51 Dry Dart). Responses are presented as mean titer ± SEM. Means with different superscripts are significantly different (*P* < 0.05).

All treatments demonstrated greater (*P* < 0.01) mean humoral responses to S2308 on the ELISA assay after experimental challenge when compared to mean responses of sera obtained prior to challenge (Figure 4). Booster vaccinates had greater (*P* < 0.05) mean ELISA responses at 4 weeks after challenge when compared to the other 2 vaccination treatments. However, mean humoral responses of all 4 treatments to S2308 did not differ (*P* > 0.05) for samples obtained at necropsy after parturition or abortion.

### Protection and Colonization After Experimental Challenge

When compared to rates in non-vaccinated bison, bison in all 3 RB51 vaccination treatments (single or booster vaccinated) had a reduced (*P* < 0.05) incidence of abortion (Table 1). In addition, bison administered single or boostered parenteral RB51 vaccinations had a reduced (*P* < 0.05) incidence of infection in uterine (placentome, vaginal swab, and/or internal iliac lymph node) and fetal samples (fetal lung, liver, spleen, gastric contents, bronchial lymph node or rectal swab). Bison booster vaccinated with RB51 also had a reduced (*P* < 0.05) percentage of animals with colonization of maternal samples as compared to the incidence of infection in non-vaccinated bison. In comparison, bison in the RB51 dry dart vaccination treatment did not differ (*P* > 0.05) from the control group in the percentage of animals in which *Brucella* was recovered from uterine, mammary, fetal, or maternal tissues at necropsy.

**Table 1:** Efficacy of *Brucella abortus* strain RB51 vaccination strategies in protecting against experimental challenge at midgestation with 10^7^ colony-forming units of *B. abortus* strain 2,308.

	**Rate of abortion or infection (Number aborted or infected/Total)**				
	**Abortion**	**Uterine**	**Mammary**	**Fetal**	**Maternal**
		**infection^a^**	**infection^b^**	**infection^c^**	**tissues^d^**
Single RB51	43% (3/7)*	43% (3/7)*	57% (4/7)	43% (3/7)*	100% (7/7)
Booster RB51	20% (1/5)*	20% (1/5)*	20% (1/5)*	20% (1/5)*	40% (2/5)*
RB51 Dry Dart	50% (3/6)*	66% (4/6)	66% (4/6)	66% (4/6)	66% (4/6)
Control	100% (7/7)	100% (7/7)	100% (7/7)	100% (7/7)	100% (7/7)

Means denoted with “*” are significantly different (P <0.05) from the control treatment.

a *Placentome, vaginal swab, and/or internal iliac lymph node*.

b *Mammary tissues (4 quarters), milk, and/or supramammary lymph node*.

c *Fetal lung, liver, spleen, gastric contents, bronchial lymph node, or rectal swab*.

d *All maternal tissues including blood and swabs*.

An efficacious vaccine would also demonstrate reduced colonization in tissues after experimental challenge. In the current study, bison receiving single or boostered parenteral vaccination had reduced (*P* < 0.05) mean *Brucella* colonization (CFU/gm) in placenta, mammary gland, various lymph nodes (mandibular, prescapular, and supramammary), and fetal tissues (lung, liver, spleen, and bronchial lymph node) when compared to mean colonization in tissues obtained at necropsy from non-vaccinated bison (Representative data in Table 2). In comparison, bison inoculated with the RB51 dry dart demonstrated a mean reduction in colonization when compared to controls in mammary gland and fetal spleen samples. When comparing to single parenteral RB51 vaccination, there was a trend for boostered vaccinates to have lower mean colonization in tissues, but differences were only statistically different (*P* < 0.05) in mammary gland samples.

**Table 2:** Log 10 colonization (colony-forming units (CFU)/gm) of S2308 in tissues at necropsy after conjuctival challenge with 10^7^ CFU of *B. aboruts* in midgestation.

	**Control**	**Single RB51**	**Boostered RB51**	**RB51 Dry Dart**
Parotid LN	2.5 ± 0.6 (6/7)	1.5 ± 0.9 (3/7)	1.1 ± 0.7 (2/5)*	2.4 ± 0.9 (4/6)
Prescapular LN	2.1 ± 0.4^a^ (6/7)	0.5 ± 0.3^b^ (2/7)*	00 ± 0^b^ (0/5)*	1.1 ± 0.7^ab^ (4/6)
Supramammary LN	3.8 ± 1.3^a^ (7/7)	1.3 ± 0.7^b^ (3/7)*	0.3 ± 0.3^b^ (1/5)*	1.5 ± 0.9^b^ (4/6)
Placentome	8.4 ± 1.2^a^ (7/7)	3.2 ± 1.9^b^ (3/7)*	2.0 ± 2.0^b^ (1/5)*	6.3 ± 2.1^c^ (4/6)
Mammary gland	1.7 ± 0.3^a^ (4/7)	0.6 ± 0.2^b^ (2/7)	0 ± 0^c^ (1/5)	0.7 ± 0.3^b^ (2/6)
Fetal Tissues				
Lung	5.2 ± 0.9^a^ (7/7)	1.0 ± 0.7^b^ (2/7)*	1.5 ± 1.5^b^ (1/5)*	4.4 ± 1.6 ^a^ (4/6)
Liver	3.6 ± 0.6^a^ (7/7)	0.9 ± 0.7^b^ (2/7)*	0.8 ± 0.8^b^ (1/5)*	2.8 ± 1.0^ab^ (4/6)
Spleen	3.7 ± 0.8^a^ (7/7)	0.9 ± 0.6^bc^ (2/7)*	0.4 ± 0.4^c^ (1/5)*	3.3 ± 1.3^b^ (4/6)

*Data are presented as mean colony-forming units (CFU) per gm of tissue and incidence of recovery (culture positive/total in treatment)*.

*Treatment means within a tissue with different superscripts are statistically (P <0.05) different*.

Incidence of recovery indicated with a “*” are different (P <0.05) from control treatment.

Incidence of infection (percentage of animals that were culture positive) in individual tissues tended to follow similar trends as colonization data. In many tissues, single and boostered parenteral vaccinates, but not animals inoculated with the RB51 dry dart, demonstrated reduced (*P* < 0.05) numbers of animals from which *Brucella* was recovered at necropsy when compared to non-vaccinated animals (Table 2). There was a trend (*P* > 0.05) for many tissues of booster vaccinates to have a reduced percentage of animals from which *Brucella* was recovered when compared to bison receiving a single parenteral vaccination with RB51.

## Discussion

Currently, there remains a need to develop a highly efficacious brucellosis vaccine that can be remotely delivered to free-ranging bison without inducing adverse responses or causing environmental concerns. Due to the difficulties associated with targeting free-ranging wildlife, an ideal vaccine would induce a high degree of efficacy with a single inoculation. The most common safety concern for live brucellosis vaccines is the observation that inoculation of pregnant animals can cause abortion and/or fetal loss. This safety concern could be addressed by implementation of a vaccination program that targets prepubescent bison or concentrating vaccine delivery during summer months when most female bison are not pregnant. At the current time, a strain and delivery method combination for bison that would meet efficacy and safety needs remains to be identified.

In the current study, we evaluated a new dry dart formulation. Theoretically, the formulation has safety benefits since it is not a liquid vaccine and therefore, should pose less risk for aerosol exposure of personnel. It also prepares the vaccine dosage in a scalable and ready to use form that could be accommodated by remote delivery systems. In addition, in a compact, lyophilized form the preparation may have a longer shelf life than liquid vaccine. Inoculation of bison with the dry dart formulation containing approximately 10^10^ CFU of live RB51 reduced abortions, but not infection in most tissues after experimental challenge when compared to non-vaccinated bison. There was a trend that efficacy of the dry dart in preventing infection was slightly less than a single parenteral dose of RB51 in the current study. As lateral transmission of brucellosis is primarily through fluids or tissues associated with parturition or abortion, the trend for the dry dart to have less infection in uterine or fetal tissues when compared to control bison could suggest potential beneficial effects in preventing disease transmission. It should be noted that abortion and infection rates in the dry dart treatment in the current study were similar to data from other experimental challenge studies of bison inoculated with a single parenteral dose of RB51 (6, 8).

The dry dart formulation appears capable of inducing humoral responses against RB51, that are statistically higher than control animals, but of lower magnitude on the ELISA as compared to bison parenterally vaccinated. Additionally, these responses are short-lived, as they are only observed at 4 and 8 weeks post-initial vaccination. Similarly, when analyzing cellular immune responses, there is a trend toward an increase in PBMC proliferation following dry dart delivery, however, this response is also short-lived as it was only observed at 8 weeks post-administration. These data would suggest that RB51 antigen was released from the dry dart and induced an immune response, yet responses were not sustained. This is reminiscent of immune responses observed with low bacterial loads or killed antigen (13–16). While bacterial counts indicate that the dry dart contained 9.9 × 10^9^ CFU of live RB51, it is possible that following implantation, bacteria were either not properly released from the pellet, or were killed prior to release, effectively resulting in a reduced vaccine dose.

While the dry dart would be a method to remotely delivery vaccine to wildlife, these data suggest that even when directly implanted, it may not be sufficient to elicit sustained, protective immune responses. We have already shown that RB51 vaccination in bison benefits from a booster inoculation to enhance immune responses and protection [(5, 17) and data from this manuscript]. Therefore, delivery of sufficient antigen and at the right time appear to be critical. The dry dart approach, however, does allow for potential modifications to the payload that may improve protective immunity but could still be incorporated into the delivery formulation. One approach might be to incorporate an immunomodulator into the formulation to enhance immunologic responses. Although there are some possible adjuvants that could be used, we are not aware of data suggesting that possible adjuvant candidates enhance immunologic responses to live vaccines targeting intracellular bacteria in large ruminants. In addition, any immunomodulating compound would most likely be required to have stimulatory activity for weeks or months to correlate with the persistence of the live vaccine strain. A second option might be preparation of a dry dart in which RB51 is incorporated into some antigen delivery platform, such as polyanhydrides. Polyanhydrides are biodegradable polymers with demonstrated safety in animals that protect incorporated antigens from degradation and can be engineered for long-term antigen release. Polyanhydrides can be engineered not only to deliver a sufficient initial dose, but also a boosting dose. Previously, vaccination of cattle with RB51 incorporated in extended release polyanhydrides primed immune responses such that *in vivo* recall responses of polyanhydride vaccinates to booster vaccination was equivalent to responses of cattle parenterally inoculated with live RB51 (18).

Data from the current study also reaffirms previous reports that boostered parenteral vaccination of bison with RB51 induces greater protective immunity than a single parenteral inoculation in preventing abortion and infection after experimental challenge. Like other studies characterizing responses in PBMC after booster vaccination, data from the current study did not find significant antigen-specific proliferative responses in circulating PBMC after a second vaccination (5, 17). It should be noted that a booster vaccination strategy will be difficult in free-ranging wildlife because of difficulties in delivery of any vaccination to these populations. Therefore, it will be essential that any vaccination strategy for wildlife populations induce optimal levels of protection after a single inoculation.

In conclusion, there currently are vaccination strategies against brucellosis that could be utilized for controlling, but not eradicating brucellosis in some bison populations. These strategies are unlikely to be feasible for all bison herds due to the need for at least two parenteral vaccinations. Although our dry dart strategy did reduce abortions in vaccinated bison, the formulation will need to be refined to enhance immunologic responses and protective immunity before it can be evaluated as a remote delivery system. However, accumulated data are encouraging that a remote delivery brucellosis vaccination strategy for bison can be developed that could not only be effectively delivered to free-ranging populations, but would be efficacious in controlling brucellosis.

## Data Availability Statement

The original contributions presented in the study are included in the article/supplementary material, further inquiries can be directed to the corresponding author/s.

## Ethics Statement

The animal study was reviewed and approved by National Animal Disease Center Animal Care and Use Committee.

## Author Contributions

All authors participated in study design, data acquisition, data analysis, and preparation of the manuscript.

## Conflict of Interest

The authors declare that the research was conducted in the absence of any commercial or financial relationships that could be construed as a potential conflict of interest.

## Publisher's Note

All claims expressed in this article are solely those of the authors and do not necessarily represent those of their affiliated organizations, or those of the publisher, the editors and the reviewers. Any product that may be evaluated in this article, or claim that may be made by its manufacturer, is not guaranteed or endorsed by the publisher.
